# Psychometric Properties of a Cultural Adapted Version of the Assessment of Identity Development in Adolescence in Panama

**DOI:** 10.3389/fpsyt.2022.806033

**Published:** 2022-03-31

**Authors:** Sergio González Flores, Kirstin Goth, Ruben A. Díaz-Hernandez

**Affiliations:** ^1^Escuela de Psicología, Universidad Santa María La Antigua, Panama City, Panama; ^2^Department of Child and Adolescent Psychiatry, University Clinics of Basel, Basel, Switzerland

**Keywords:** identity, Criterion A, cultural adaptation, personality, adolescence

## Abstract

The Assessment of Identity Development in Adolescence (AIDA) is a self-report instrument to detect pathological development of Identity. In Panamá, psychometric instruments for assessment of psychopathology in adolescence are lacking. Our aim was to develop a valid and reliable version of the AIDA Inventory for Panamanian Population. AIDA was adapted to Spanish considering cultural aspects of Panamanian population. Two pilot tests were performed prior to main test to assess item-total correlation at subscale, primary scale and total scale levels and internal consistency at subscale level. A mixed sample of students and PD patients (*N* = 315) completed the AIDA inventory, the “Strength and Difficulties Questionnaire” and “Defense Style Questionnaire−40.” AIDA was retested in a sub sample from school population (*n* = 98). The Structured Clinical Interview for Axis II Disorders was used for diagnosis of personality disorders in the patient sample (*n* = 25). Psychometric properties were tested to assess internal consistency, reliability, factorial validity, convergent validity, and criterion validity. AIDA Panama showed excellent internal consistency for the total scale Identity Diffusion with Cronbach's α:0.94 and a retest reliability of 0.84. A Bifactorial CFA was modeled to assess the dimensionality of the inventory. The proportion between OmegaH and Omega at total scales 96% of the variance is explained by a general factor. Furthermore, the Explained Common Variance for the General Factor is 73% supporting unidimensionality. In line with theory, AIDA total scale showed a high positive correlation (*r* = 0.67) with Total Difficulties scale and high positive correlation (*r* = 0.71) with Immature Defense scale. The AIDA total score differed highly significant (*p* = 0.000) between the patient sample and the students with a large effect size (*d* = 1.02).

**Conclusion:**

The adaptation and validation of AIDA for Panamanian adolescent population was successful with good psychometric properties and significant correlations with related psychopathological constructs. AIDA showed high clinical validity by providing a valid discrimination between the school sample and a diagnosed PD sample, in line with the assumption that impaired identity functioning is at the core of personality disorders, especially in adolescence.

## Introduction

The Assessment of Identity Development in Adolescence (AIDA) is a self-report inventory with an integrative focus between identity development and impairment in personality functioning for adolescents aged between 12 and 18 years (1). It was developed by a Swiss-German-American research group, inspired by the alternative model of personality disorders (AMPD) from section III in DSM-5 (2), emphasizing the severity of impairments in personality functioning with other clinical concepts of identity pathology with a target on the complex relationships between identity development and the vulnerability for developing personality disorders (3). The personality functioning approach can also be found in the ICD-11, where the diagnosis of personality disorders has transitioned to a dimensional model (4) considering Self functioning as an important domain on personality with empirical support and the accessibility to diverse measures for its assessment (5). Moreover, Identity as a function is considered in assessment for treatment plans in the Operationalized Psychodynamic Diagnosis (6) and the Psychodynamic Diagnosis Manual−2 (7). The assessment of identity and Self in these diagnostic systems has advantages like the inclusion of children and adolescents if there are evidence of impairments in personality development. The early diagnose of these disorders is a priority in mental health since this pattern could become more stable in adulthood and their severity be identified with early intervention in adolescence (8, 9). Furthermore, mental health interventions assess identity as an outcome for treatment (10) specially in psychotherapy models for personality disorders as mentalization based treatment (11), transference focused psychotherapy (12), dialectical behavioral therapy (13), and identity adolescent treatment (14).

Impaired identity development is seen as one of the relevant domains of personality functioning and as a core marker of Personality Development, especially in adolescence (15–17). From a psychodynamic perspective, Otto Kernberg's Personality Organization model (18) describes Borderline Personality structured by Identity Diffusion along with Primitive Defenses and Impaired reality testing (12). Defense mechanisms in borderline personality organization are based on splitting, reflecting lack of integration in self and displaying other defenses as projection, denial, and projective identification that keeps the split mechanism on mental representations within self and significant others (14, 19). In empirical research, this concept is operationalized as defense styles and differentiates between healthy population and clinical population (20). Defense mechanisms are automatic and implicit responses with a significant role in adaptation and regulation (21, 22).

In emerging personality disorders, difficulties in psychosocial adjustment as emotional regulation (23) identification of affects and feelings (15, 24) and psychiatric symptoms from the internalizing (25) and externalizing spectrum are often found in personality and identity pathology (26) representing a significant risk in adolescence to establish personality disorders and interfering with healthy development (27).

### Assessment of Identity Development in Adolescence

The construction of the inventory AIDA followed basic principles of analyzing developmental psychopathology (28) starting with defined theory-based model of identity, integrating the relevant subconstructs concerning pathological identity development regarding a broad range of theoretical descriptions and empirical results in social-cognitive and psychodynamic theories considering operationalizations of adaptive and maladaptive identity development by authors like Kernberg (18), Eriksson (29), James (30), Livesley (31), Westen (32), Akhtar and Samuel (33), and Bateman and Fonagy (34). Based on this, the basic AIDA model was formulated with the two domains “Continuity” and “Coherence,” further subdivided in psychosocial areas of functioning as, self-related, social related and mental representations, building the higher order dimension “Identity Integration vs. Identity Diffusion.”

The first validated test version of AIDA was in German language (1) proving sound psychometric properties on internal consistency, exploratory factor analysis and discrimination between clinical population and healthy controls. In a clinical study (35), the AIDA scores showed adequate capacity to discriminate between patients with externalizing, internalizing, and personality disorders with the latter showing the highest scores among the groups and externalizing disorders the lowest (35). Actually, the AIDA has several cultural adaptations (see https://academic-tests.com) with very similar results regarding reliability, intercorrelations of the scales, and factor structure according to principal component analysis as an exploratory method. Exploratory Structural Equational Modeling has been used to test factorial validity in Chilean population (36) and Italian population (37), with both studies assessing a bi factorial structure with one general factor and six specific factors regarding the subscales of the inventory, with results that support unidimensionality of the inventory with better fit than other analyses like Confirmatory Factor Analysis. Moreover, the AIDA across diverse cultures showed evidence of clinical validity by differentiation between healthy and clinical or at risk populations, as shown with the Chilean and Italian validation studies. In Mexico (38), the inventory was tested with adolescents engaged on criminal activity, finding significant results in comparison with healthy controls. In Brazilian population (39), with adolescents reporting psychiatric symptoms with the Strength and Difficulties Questionnaire and impairment on reflective functioning (34), findings were significant differences with adolescents who reports better psychological adjustment. In Austrian population, significant differences were found between adolescents with internet addiction and problematic internet use and healthy controls (40).

The adaptation and validation of measures from a dimensional model to detect emerging personality disorders in their different domains is an important task in child and adolescent mental health research (8). Tools for screening mental health issues during adolescence enables early diagnosis and treatment of psychological vulnerabilities that would, otherwise, may show transition into complex personality disorders in adulthood (9, 27, 32). The adaptation and validation of accessible dimensional measures is necessary for prevention of pathological development (41). To our knowledge, the adaptation and validation of psychometric measures for adolescent population in Panamanian population is lacking. The cultural adaptation of the AIDA inventory for Panamanian Adolescent Population could be beneficial for researchers as well as for clinicians for diagnostic purposes.

The present study aims to test the psychometric soundness of a culture adapted Spanish version of the AIDA for Panamá. We set a special focus on testing factorial validity by using bi factor models with a general factor and six specific factors according the AIDA subscale level. Another focus is at the detailed convergent and discriminant validity, investigating the relations between Identity functioning and Defenses Styles and psychopathology.

## Methods

### Participants and Procedures

The school sample assessment was selected by convenience and performed at one private school from an urban area in Panamá City. We extended an invitation to all high school students explaining the purpose and procedures of the study, enclosing informed consent, and assent forms for them and their parents. From a total of *n* = 500 sent invitations, we only included in the assessement the *n* = 295 students who returned both consent and assent forms signed. The time for the first assessment took between 20 and 30 min in which students had to respond three self-report measures: AIDA, the Defense Style Questionnaire (DSQ), and the Strength and Difficulties Questionnaire (SDQ). Two weeks later, the AIDA was retested in a 10–15-min session by *n* = 98 of the participants.

The clinical sample recruitment was performed at “*Cl*í*nica Psicológica de Terapia Familiar,”* an outpatient University treatment facility that offers psychotherapy for adolescents. The assessment was made with patients on waiting list, not receiving psychotherapy or psychopharmacology treatment, and not displaying psychotic symptoms. Reasons for consultation included low academic performance, feelings of emptiness, anger management issues, depressive feelings, suicidal thoughts, or non-suicidal self-injury, and low self-esteem. We approached adolescents and their parents to explain the purpose of the study. Those who agreed to participate were required to sign informed consent and assent forms. From the 35 families approached, a total of 20 families agreed for participation on the study. The assessment was conducted in two sessions: during first session the Structure Clinical Interview for Axis II Disorders (SCID II) was administered; at the second session, the adolescents completed three self-report measures: AIDA, DSQ, and SDQ.

The total sample of 315 participants (142 boys, 173 girls; mean age of 14.9, SD 1.7) consisted mainly of the students with *N* = 295 participants (131 boys, 164 girls; mean age of 14.9, SD 1.7). The school sample was enriched by selected *n* = 20 patients diagnosed with Personality Disorder_to include also impaired participants with assumed higher levels of the targeted constructs in the analyses and being able to interpret the results toward pathology. The sample size achieved allows us to identify replications on our results regarding the original german study (1) and Mexican study (38).

The clinical sample included 20 participants (11 boys, 9 girls) with a mean age 14.9 (SD 1.7). According to the SCID II interviews, half of participants from this sample met criteria for two personality disorders and the other half for one personality disorder. Borderline personality disorder was the most frequent diagnosis found on 75% (*n* = 15) of the clinical sample. Other diagnosis found were avoidant personality disorder (*n* = 7), narcissistic personality disorder (*n* = 4), obsessive compulsive personality disorder (*n* = 2), and antisocial personality disorder (*n* = 1).

### Scale Adaptation

In the first step, the AIDA was culturally adapted for Panama in cooperation with the original authors. The cultural adaptation process on item formulation focused on content equivalence regarding appropriate language for young people and culture-appropriate disease related behaviors. Standardized procedures of culture-adapted test construction were followed, in reference to the guidelines of the International Test Commission (42), including step-by-step item optimization based on empirical beta, pilot, and main tests using mixed samples with both students and patients showing relevant features of the pathology that is supposed to be investigated with the developed assessment tool, in order to have the full variance of the targeted construct in the data. The original authors performed the statistical calculations to ensure equivalent standards in the methods.

### Measures

#### Assessment of Identity Development in Adolescence (AIDA)

The AIDA (1, 43) has fifty-eight multiple-choice items with a five-point scale response ranging from 0 (“Strongly disagree”) to 4 (“Strongly agree”). The total scale “Identity Diffusion” consists of two primary scales “Continuity” and “Incoherence.” High scores are speaking for high impairment in identity functioning. Each primary scale has three subscales each. Original study reported high internal consistency Cronbach's Alpha with 0.94 for the total diffusion scale 0.87 for the discontinuity scale and 0.92 for the incoherence scale and from 0.69 to 0.84 for the subscales. The inventory can be found in several translated versions on the project website https://academic-tests.com and can be requested for free for research studies.

#### Defense Style Questionnaire (DSQ 40)

The Defense Style Questionnaire (44) was developed for adults and has forty items that assesses 20 defense mechanisms grouped in two item paired scales forming major order scales of three factors: mature, neurotic, and immature defenses according to Vaillant's model of Ego Defenses in psychoanalytic theory. The DSQ-40 on adolescent population is an appropriate measures with good psychometric properties (45). We used a Spanish version from Mexico (46). In the present study, Cronbach's Alpha coefficients for the scales were 0.77 for immature defenses 0.38 for neurotic defenses and 0.41 for mature defenses.

#### Strengths and Difficulties Questionnaire—SDQ

The Strength and Difficulties Questionnaire (47) is a screening tool with 25 items grouped in four difficulty scales measuring emotional problems, peer problems, conduct problems, and hyperactivity and one strength scale measuring prosocial behavior. This questionnaire is used to differentiate normal population from clinical population in terms of emotional and behavioral symptoms in children and adolescents (47). In this study, the self-report format has been used which is appropriate for ages from 11 to 17. We used the Spanish translation of the test that can be found at the official website (www.sdqinfo.com). In the present study, Cronbach's alpha coefficient were 0.68 for total scale, 0.62 for prosocial scale, 0.60 for hyperactive scale, 0.67 for emotional problems scale, 0.46 for conduct problems scale, and 0.47 for peer problems scale.

#### Structured Clinical Interview for Axis II Disorders—SCID II

This Structured Interview (48) is designed to diagnose Personality Disorders according to DSM criteria. It has a self-report instrument with 119 items using a Yes/No format for responses, and 119 questions for the Interview. Items answered as Yes in the self-report instrument are explored in the Interview. The interview uses a 3-response format, 1 meaning absence of criteria, 2 subclinical criteria and 3, present criteria. Although it is developed for adults, it is frequently used also in adolescents, internationally (49).

### Statistical Analysis

We used SPSS 24 (50) and R (32) with lavaan (51) and bifactorindicescalculator (52) packages for data analysis. Basic psychometric properties were evaluated with the full combined sample of *n* = 315 students and patients. Item analyses and selection was based on the following criteria: percentage of symptomatic answers (p_it_ 5–95%), percentage of missingness (<10%), partial eta square as a measure of the effect size of gender- or age-related item bias ($\eta_{\text{p}}^{2}$ > 0.14), and item-total correlation r_it_ > 0.30. For translated inventories, the criteria can be set to r_it_ > 0.20 as well but mean r_it_ should at least not be <0.10. The mean r_it_ was built of the results referring to the subscale, the primary scale, and the total scale.

Scale level analyses included internal consistency, retest reliability, and factorial, construct, and criterion validity. Internal consistencies were evaluated by Cronbach's alpha and were supposed to exceed 0.80 at total scale level, 0.70 at primary scale level, and 0.60 at subscale level as adequate for heterogeneous contents, while homogeneity coefficients α > 0.80 would be very good and >0.90 excellent. Retest reliability was calculated with Pearson correlation and their 95% confidence interval.

Factorial Validity was assessed with bi factorial Confirmatory Factor Analysis to evaluate the model of a general factor—Identity diffusion—and six specific factors, referring to the subscales. In previous studies, Bi factor models has shown better fit than traditional CFA models (36, 37). The model parameters were computed using maximum likelihood estimation and the model fit was evaluated with traditional cut off values, expecting above 0.90 for Comparative Fit Index, above 0.90 for Tucker Lewis Index, below 0.08 for Root Mean Square Error Approximation and below 0.06 for Standardized Root Mean Square Residual (53, 54). The bifactorial Confirmatory Factor Analysis has been criticized for overfitting models (55). However, further tests to analyze and understand the factorial validity in Bifactorial confirmatory factor analysis are suggested to avoid bias on fit criteria (56). We included assessment for reliability with McDonald's omega (57). Furthermore, to find if the general factor identified accounts for the majority of variance we tested the proportion of Omega and Omega Hierarchical expecting values over 0.80 (58–60) and the explained common variance (ECV) on the general factor and the specific factors expecting to be over 60% (61). At last, we calculated the Proportion of uncontaminated correlations (PUC) and relative bias (58, 59).

Construct validity in terms of convergent and discriminant validity was checked by correlation analysis with Pearson r coefficient relating the AIDA scores with SDQ scores and the DSQ−40 scores.

Criterion validity was analyzed by means of a Welch's *t*-test (62) comparing the AIDA scores between the clinical and the school sample. We calculated Cohen's d as a standardized measure of effect size to deal with big differences in sample size and for a better intuitive interpretation of the results, as *d* = 1 corresponds to the familiar unit “1 standard deviation” to describe a difference (63). We expected to reach a large difference (*d* > 0.80) to avoid over-interpretation and artificial establishing of developmental differences.

To test for systematic differences on gender and age in the levels of identity diffusion we compared the AIDA scores between boys and girls and between different age groups of the school sample. Differences concerning age were tested for the full factor age and additionally divided into the age groups of early-to-middle (12–14 years) and middle-to-late (15–18 years) adolescence in accordance with the procedure used for the original version of *AIDA*. All group comparisons were performed with the raw scores using MANOVA (multivariate analysis of variance). Score differences were examined concerning significance (1% level) and effect size. The relevant statistical parameter for the evaluation of meaningful group differences in MANOVA is the effect size “partial eta square” ($\eta_{\text{p}}^{2}$) with $\eta_{\text{p}}^{2}$ > 0.01 (small effect), $\eta_{\text{p}}^{2}$ > 0.06 (medium effect), and $\eta_{\text{p}}^{2}$ > 0.14 (large effect).

This study was conducted with approval by the ethics committee of Hospital Santo Tomás in Ciudad de Panamá. All participants and their parents were informed about the purpose of the research, data confidentiality and anonymization via an explanatory document signed—upon agreement—informed assent and consent forms for the study.

## Results

### Reliability

All 58 items matched the criteria for percentage of symptomatic answers as a sign of a balanced response pattern. No item showed a high rate of missingness and therefore no sign of systematic problems to answer the item. All 58 items showed “item fairness” as no systematic differences with remarkable effect sizes in the responses according to gender and age were detected. Calculations of the mean for item-total correlation was between 0.3 and 0.6 for 56 items and between 0.2 and 0.3 for two items.

Internal consistencies met the criteria with Cronbach's Alpha for the total scale Identity-Diffusion with 0.94, for the two primary scales Discontinuity with 0.85 and Incoherence with 0.91, as for the subscales scores were ranging from 0.65 to 0.80. The 2-week retest reliabilities analyzed with pearson correlation coefficient were good with 0.84 for the total scale Identity-Diffusion, for the two primary scales Discontinuity with 0.73 and Incoherence with 0.77, as for the subscales scores were ranging from 0.63 to 0.77 (see Table 1).

**Table 1:** Internal consistency Cronbach's α for the total scale, the primary scales, and the subscales of AIDA Panama in the mixed sample *n* = 315.

**Scale**	**No. items**	**Main test Cronbach's α** ***n* = 315**	**Retest reliability** ***n* = 98**	**95% Confidence interval**
**Identity diffusion**	**58**	**0.94**	**0.84**	**0.78–0.90**
**Discontinuity**	**27**	**0.85**	**0.73**	**0.65–0.83**
Perspectives	9	0.65	0.73	0.63–0.82
Relationships	11	0.77	0.63	0.54 −0.77
Emotional self-experience	7	0.75	0.67	0.60–0.80
**Incoherence**	**31**	**0.91**	**0.82**	**0.80–0.91**
Consistency	11	0.80	0.77	0.69–0.85
Autonomy	12	0.80	0.77	0.75–0.88
Cognitive self-experience	8	0.75	0.69	0.60–0.80

*Retest reliability in a school subsample (n = 98). Bold letters and numbers were put to distinct the total scale (Diffusion) and primary scales (Incoherence, Discontinuity) and their correspondent coefficients values*.

### Factorial Validity

The fit indices for the confirmatory bi-factor model (a general scale and six sub-scales) showed a mixed picture (see Table 2). The incremental fit indices, Comparative Fit Index and Tucker Lewis Index, and one of the absolute fit indices, Standardized Root Mean Square Residual, did not met the established criteria, while Root Mean Square Error Approximation did (53).

**Table 2:** Descriptive fit indices of bifactorial confirmatory analyses.

**Model**	**Parameters**	***x* ^2^**	**df**	**CFI**	**TLI**	**RMSEA**	**RMSEA 90%** **confidence interval**	**SRMR**
Bi factorial CFA (1g + 6s)	231	2965.31	1,538	0.77	0.752	0.051	0.048–0.054	0.098

*df, degrees of freedom; CFI, Comparative Fit Index; TLI, Tucker- Lewis Index; RMSEA, Root Mean Square Error of Approximation; SRMR, Standardized Root Mean Square Residual*.

In the ECV analysis, several bi-factorial indices for the general scale and the subscales were compared (see Table 3). Coefficient Omega was high for the general factor. When we compare the Omega hierarchical (ω_H_ = 0.91) with the omega (ω = 0.95), most of the variance in total scores (ω_H_/ω = 95.8%) is attributed to the general factor. The ECV for the general factor was strong (0.73) and the PUC was high (0.84), thus indicating that one can reliably conclude that the common variance is essentially unidimensional. All subscales showed high omega scores. However, when controlling for the variance attributed to the other subscales in omega hierarchical, none of the subscales showed adequate results. Consistency subscale explained the least percentage of variance in total scores (ω_H_/ω = 21.0%); while Perspective subscale, the largest (ω_H_/ω = 43.0%). On the other hand, the general factor can explain a large percentage of the variance of items in each subscale (ECV gs): ranging from 0.40 for the Perspective subscale to up to 0.89 for the Consistency subscale. The Consistency scale has the lowest ECV ss (proportion of common variance of the items in a factor which is due to that factor). All but two of the subscales (Perspective and Consistency) showed adequate factor determinacy scores. As such, we cannot be confident that the individual differences on the factor score estimates for these subscales are good representations of true individual differences on the factor. Construct replicability (H) was low in all but two of the subscales (Perspective and Autonomy). This means that caution must be exerted when interpreting regression paths between these factors and other latent variables. Taken together, this results show the Perspective and Autonomy subscales have better properties than the rest (58, 59).

**Table 3:** Reliability indices for bifactor confirmatory analysis.

**Factors**	**Omega ω**	**Omega ω H**	**ECV** **ss**	**ECV** **sg**	**ECV** **gs**	**PUC**	**Relative bias**	**FD**	**H**
**Diffusion**	**0.95**	**0.91**	**0.73**	**0.73**	**0.73**	**0.84**	**0.05**	**0.98**	**0.96**
Perspectives	0.72	0.41	0.60	0.08	0.40			0.68	0.68
Relationships	0.73	0.14	0.27	0.04	0.73			0.77	0.53
Emotional	0.82	0.07	0.23	0.04	0.77			0.79	0.51
Consistency	0.85	0.02	0.11	0.02	0.89			0.67	0.32
Autonomy	0.84	0.21	0.29	0.06	0.71			0.81	0.59
Cognitive	0.81	0.11	0.23	0.04	0.77			0.73	0.48

*ECV ss, ECV of a specific factor with respect to itself; ECV sg, ECV of a specific factor with respect to the general factor; ECV gs, ECV of the general factor with respect to a specific factor; PUC, percentage of uncontaminated correlations; FD, factor determinacy coefficient (ρ); H, construct replicability*.

### Convergent and Discriminant Validity

The AIDA total score showed a high positive correlation of 0.67 with the SDQ total score (see Table 4), both assumed to represent pychopathology. The AIDA primary scales and subscales showed very similar patterns, also concerning the SDQ primary scales of emotional problems, conduct problems, and peer problems. Moreover, the AIDA total score showed a high positive correlation of 0.71 with the DSQ-40 Immature Defenses scale, which is supposed to denote pathological defense mechanisms. Again, the AIDA primary scales and subscales showed very similar patterns. The further DSQ-40 scales Mature Defenses and Neurotic Defenses had low correlations with the AIDA scores showing coefficients of −0.11 and 0.03, respectively.

**Table 4:** AIDA scale correlations with Strength and Difficulties Questionnaire and Defense Style Questionnaire - 40.

	**Strength and difficulties** **questionnaire**						**Defense style** **questionnaire - 40**		
	**Total** **scale**	**Prosocial**	**Hyperactive**	**Emotional** **problems**	**Conduct** **problems**	**Peer** **problems**	**Immature** **defense** **style**	**Neurotic** **defense** **style**	**Mature** **defense** **style**
**Diffusion**	**0.67**	**−0.148**	**0.272**	**0.558**	**0.374**	**0.429**	**0.706**	**0.039**	**−0.111**
**Discontinuity**	**0.62**	**−0.128**	**0.215**	**0.547**	**0.323**	**0.426**	**0.636**	**−0.016**	**−0.167**
Perspectives	0.33	−0.160	0.106	0.313	0.159	0.221	0.365	−0.109	−0.270
Relationships	0.58	−0.165	0.183	0.488	0.344	0.420	0.596	−0.048	−0.125
Emotional self experience	0.53	0.029	0.218	0.487	0.243	0.352	0.531	0.120	−0.015
**Incoherence**	**0.642**	**−0.149**	**0.288**	**0.518**	**0.377**	**0.394**	**0.693**	**0.076**	**−0.062**
Consistency	0.589	−0.77	0.244	0.454	0.379	0.380	0.646	0.023	−0.028
Autonomy	0.547	−0.058	0.273	0.466	0.285	0.304	0.569	0.119	−0.084
Cognitive self experience	0.575	−0.174	0.248	0.457	0.342	0.369	0.630	0.056	−0.050

*r > 0.10 low correlation; r > 0.30–0.50 moderate correlation; r > 0.50 high correlation. Bold letters and numbers were put to distinct the total scale (Diffusion) and primary scales (Incoherence, Discontinuity) and their correspondent coefficients values*.

### Criterion Validity

To analyze the criterion validity of AIDA, which is the central psychometric criteria for a pathology-related instrument, we compared the AIDA scale and subscale scores between the school sample and the clinical PD patient sample (Table 5). The AIDA total score differed highly significant (*p* < 0.001) between the PD-group and the students with a large effect size of *d* = 1.02 standard deviations (>0.80 = large effect). The AIDA subscales showed similar patterns except subscale 1.1 “Discontinuity concerning attributes, talents, perspectives” which showed no significant discrimination between the healthy and the impaired sample.

**Table 5:** Differences in AIDA mean scores (mean) and standard deviations (SD) between students and PD patients; significance (p) and effect size Cohen's d.

	**Students** ***N*** **=** **295**		**PD patient sample** ***N*** **=** **20**			**Effect size**
	**Mean**	**SD**	**Mean**	**SD**	***P*-value**	**Cohen's d**
**Diffusion**	**88.2**	**33.3**	**121.6**	**26.0**	**0.000**	**1.02**
**Discontinuity**	**37.5**	**14.6**	**50.3**	**13.1**	**0.000**	**0.88**
Perspectives	12.8	5.5	13.5	4.1	0.626	0.13
Relationships	11.5	6.9	18.5	9.2	0.000	0.99
Emotional Self Experience	13.1	5.8	18.4	4.7	0.000	0.93
**Incoherence**	**50.7**	**20.5**	**71.3**	**16.6**	**0.000**	**1.02**
Consistency	18.7	8.4	26.6	6.7	0.000	0.95
Autonomy	18.2	8.5	24.8	8.2	0.001	0.78
Cognitive Self Experience	13.8	6.3	19.9	6.1	0.000	0.97

*Effect size: d >0.20 small, >0.50 medium, >0.80 large. Bold letters and numbers were put to distinct the total scale (Diffusion) and primary scales (Incoherence, Discontinuity) and their correspondent coefficients values*.

### Systematic Differences According to Gender and Age

Data showed a sufficient normal distribution of the scores with values for skewness and kurtosis around 1 in the full sample. We compared the AIDA Panama scores between boys and girls and between different ages in the school sample to establish population norms. No significant group differences were found for the factors gender and age on 1% level in their levels of identity diffusion (see Table 6) on total and primary scale level.

**Table 6:** Differences in AIDA mean scores (mean) and standard deviations (SD) between younger and older adolescents and between boys and girls in the school sample significance (p) and effect size partial eta-square ($\eta_{\text{p}}^{2}$) of the differences.

	**Gender**						**Age**					
	**Male** ***n*** **=** **131**		**Female** ***n*** **=** **164**				**12–14 years** ***n*** **=** **122**		**15–18 years** ***n*** **=** **173**			
	**Mean**	**SD**	**Mean**	**SD**	**P**	** $\eta_{\text{p}}^{2}$ **	**Mean**	**SD**	**Mean**	**SD**	**p**	** $\eta_{\text{p}}^{2}$ **
**Diffusion**	**86.9**	**31.6**	**89.3**	**34.7**	**0.536**	**0.001**	**87.5**	**35.4**	**88.7**	**31.9**	**0.746**	**0.000**
**Discontinuity**	**35.9**	**13.2**	**38.8**	**15.6**	**0.090**	**0.010**	**37.1**	**14.8**	**37.8**	**14.5**	**0.682**	**0.001**
Perspectives	12.1	5.3	13.4	5.5	0.048	0.013	12.5	5.2	13.1	5.7	0.393	0.002
Relationships	11.0	6.2	11.9	7.5	0.230	0.005	11.5	7.5	11.5	6.6	0.995	0.000
Emotional self experience	12.8	5.4	13.4	6.1	0.335	0.003	13.0	6.0	13.2	5.6	0.814	0.000
**Incoherence**	**51.0**	**20.1**	**50.5**	**20.9**	**0.844**	**0.000**	**50.4**	**22.2**	**51.0**	**19.3**	**0.815**	**0.000**
Consistency	18.9	7.8	18.5	8.9	0.709	0.000	17.9	8.7	19.2	8.2	0.215	0.005
Autonomy	18.3	8.8	18.2	8.2	0.868	0.000	18.5	9.0	18.1	8.1	0.643	0.001
Cognitive self experience	13.8	6.3	13.9	6.3	0.937	0.000	14.0	6.9	13.8	5.9	0.789	0.000

*Effect size $\eta_{\text{p}}^{2}$ > 0.01 small, >0.06 medium, >0.14 large. Bold letters and numbers were put to distinct the total scale (Diffusion) and primary scales (Incoherence, Discontinuity) and their correspondent coefficients values*.

## Discussion

Our goal was to provide a culture-adequate and age-adequate assessment tool to support early detection of personality disorders in adolescence. Following strict guidelines of test construction, we adapted the AIDA original version for Panama. The AIDA is a self-report questionnaire for adolescents from 12 years up (± 2 years) to assess impaired identity functioning in line with the new dimensional severity models to diagnose personality disorders in the AMPD / DSM-5 and ICD-11 (Criterion A). The version AIDA Panama showed good scale reliability and construct validity, reasonable factorial validity, and excellent clinical validity. Nation specific population T-norms enable the use for individual diagnostics.

### Cultural Adaptation

Our first aim in this study was the cultural adaptation of the AIDA inventory to Panamanian Spanish language for adolescents. In a step-by-step process with two pilot tests the items were checked empirically and the wording was improved to have the final version for the main test. In the main test, most items had moderate levels of item total correlation, at total scale, primary scale, and subscales, suggesting sufficient associations between the content of the items in order to justify the use of sum scores on the different levels. We also found excellent levels of internal consistency with Cronbach's alpha that supports the thorough adaptation process with comparable results to other versions in Latin American adaptations (36, 38) as well as in other languages (37, 64). Inventories for countries with related cultures and similar languages, as happen in Latin America where Spanish is the dominant language, requires versions that are comprehensible for population and their cultural expressions (65). The AIDA inventory has different versions in Hispanic countries to avoid cultural bias since expressions have different meanings (66). Moreover, cultural adaptations with equivalent versions regarding the conceptualization of construct enables more reliable comparisons for analysis as measurement invariance (67, 68).

The short retest reliability (interval was 2 weeks) in the school sample shows a good stability of the assessed scales, justifying the use in tems of traits. However, the formulations of the AIDA items are focusing on the present (the last weeks) in order to enable the measurement of changes over time, e.g., for using the instrument as an outcome measure in therapy studies or developmental longitudinal studies (1).

### Factorial Validity

We analyzed the factorial validity of the AIDA considering a bifactor model with one general, Identity diffusion factor, and six specific factors corresponding to the sub-scales. The fit indices on the bifactorial confirmatory analysis were below the expected considering the traditional cut off points (53). However, conditions as the sample size, degrees of freedom, number of items, factors reliability, and the complexity of the model in study are influences over the fit indices that doesn't correspond to the fixed cut off fit indices (69–71). A closer look to the factor loadings (see Supplementary Table 1) shows that the general factor had higher loadings and fewer negative, insignificant estimates, than the sub—scales factors. The proportion of Hierarchical Omega and Omega from the subscales shows that Diffusion factor accounts for most variance and ECV coefficients support this, following a cautious suggestion for percentage above 70% (61). The Proportion of uncontaminated correlations of 84% indicates that bias of introducing an unidimensional models is trivial (58). Finally, the absolute relative bias in factor loadings between the general factor of a bifactor model and a unidimensional model is 0.05, supporting a unidimensional structure (59).

This result remarks the complexity of the Identity concept, in studies with a developmental background the dimensions of coherence and continuity tends to be studied separately (72) while referring to the same concept (73). For example, the factorial structure of the Inventory of Personality Organization via Exploratory Structural Equational Modeling has identified four factors approached as facets of identity (74), with a factor “Instability of Self and Others” as a general factor for self and interpersonal functioning that is not clearly interpretable despite showing consistency across studies. On the other hand, The Severity Indices of Personality Problems treats continuity and coherence dimensions from a self-related level of functioning and, from the coherence dimension, on social related functioning regarding the autonomy factor from the AIDA model (1, 75, 76). Lastly, the Identity Disturbance questionnaire (77) emphasizes on continuity on self—related functioning and, on the continuity dimension, the relationship factor from AIDA (1, 77). Most inventories, refers to these two dimensions across their distinct definitions, from a self-related level of functioning while other levels of functioning as social related, are more focused on a coherence dimension on the Severity Indices of Personality Problems and continuity dimension on Identity Disturbance Questionnaire. However, our findings suggest that, regardless of the definitions, the construct is the same. Nevertheless, the consideration of distinct facets of this construct is important for clinical descriptions (78).

### Construct Validity

Altogether, the AIDA Panama scales showed covariations with related constructs matching the expected assumptions.

The AIDA scales showed high correlation with Emotional problems referring to internalizing symptoms as worries, sadness, anxiety, and somatic complains. As show in previous studies, emotional difficulties like identifying affects within oneself, from narrative identity perspective (15), and being able to regulate emotions (23) are evidenced in personality and identity pathology. A similar pattern was found in peer problems scale related to interpersonal difficulties, which was expected since personality pathology is characterized for impairment in interpersonal functioning with difficulties on developing healthy and stables relationship, due to unavailability to understand oneself and understanding others (34).

In our study, Conduct problems and hyperactive scales had positive associations with lower pearson correlations coefficients than the internalizing scales. In a study performed in swiss psychiatric sample, patients with internalizing symptomatology showed higher scores (T value Diffusion scale = 69) than patients with externalizing symptoms (T value Diffusion scale = 49) (35). Our findings suggests that Identity diffusion has more association with internalizing symptoms that externalizing symptoms. In the Hungarian version of the inventory similar results were found (64) and in Turkish version of the Levels of Personality Functioning (79). The SDQ is based on a traditional symptom model and these associations are an indicator that assessing personality functioning with the AIDA inventory, for diagnosis of personality disorders, it is also related to traditional psychiatric symptoms. Moreover, this indicate that the culturally adapted version of AIDA identifies these psychiatric symptoms from new dimensional perspective that allows clinicians to have a more functional understanding of adolescents with impairments in personality development (27).

On the other hand, prosocial scale showed low negative correlations, showing a weak and inverted association with empathic and social sensitive behaviors suggesting that is a distinct construct with identity diffusion. In the German study, the AIDA scales had low negative correlations with Cooperativeness, sharing similar contents in their definitions (1).

In Otto Kernberg's theory (12, 18), borderline personality organization displays Identity Diffusion with primitive defenses mechanisms. In this study, identity diffusion scale showed high significant correlations with immature defense style as projection, acting out, and somatization. This suggests that immature defenses are present with different identity related constructs that are not present in Kernberg's personality organization model. Furthermore, the two factors called Instability of self and others and Instability of behaviors identified on the Inventory of Personality Organization displays how identity diffusion and immature defenses tends to merge (74). This result was consistent in Italian population (80), German population (81), and adolescent population (82, 83). Mature defenses as, humor and anticipation, and neurotic defenses styles as, idealization and reactive formation, had low correlations with the AIDA scales. These group of defenses in Kernberg's model correspond to neurotic and healthy level of organization (12) with complex and unconscious processes, as repression from traditional psychoanalytic theory, which might not be observable, neither to the subject or a rater, through a self-report measure (84).

### Clinical Validity

We examined differences between school population and clinical population interviewed with Structure Clinical Interview for Axis II Disorders to evaluate the ability of the AIDA version in Panama for diagnosis of Identity Diffusion. The school sample and the clinical sample differed between their total scores with a large effect size. In earlier studies, the size effect with clinical sample were higher in Mexican population with *d* = 0.84 (38) and German population with *d* = 2.17 (1) while our results are more similar to Italian population with *d* = 1.5 (37).

The AIDA subscales showed similar patterns except for subscale “Discontinuity concerning attributes, talents, perspectives” which, against our assumptions, showed no significant discrimination between the healthy and the clinical sample. The pathological impact of this aspect concerning an impaired identity development does not replicate with the version AIDA Panama. The theorical foundations of this subscale are related to Livesley “lack of continuity” (85, 86) and Eriksson “subjective self-sameness” (29) where adolescents present a diversity of roles and activities while being able to recognize themselves in distinct roles and activities. In other translated AIDA inventories this scale showed the lowest internal consistency among the subscales (1, 38). Also, this subscale has the most inverted items referring to healthy development in their wording, that might prevent bias in subjects responses but raises probability of error in measurement (87). Moreover, in other inventories like, e.g., the Inventory of Personality Organization, this construct is represented with fewer items, which makes its assessment more scarce (74). In observant rated inventories, alpha coefficients are higher in scales with related contents as stabilizing goals, perspective on future and stabilizing values (32). Self-reports and informant reports measures have discrepancies (88), part of measurement error calculated on internal consistency could be attributed on the difficulty to observe own behaviors from the outside as a failure on mentalizing capacities (34). Self-reports instruments are an economic resource for assessment, especially in complex pathology in personality disorders. It is important to approach this assessment from different resources in an integrative way combining clinical observation, self-report, and third-party information to perform valid diagnosis.

According to the results on scores' distributions, differentiated population norms according to gender and age are not necessary for Panama, matching the results for all other international AIDA versions. Pathological identity development does not seem to vary linear with gender or age (32).

### Limitations

This study has important limitations. First, our data is not perfectly representative of Panamanian population since the assessment consisted of participants from an urban area in Panama City. We consider that sociodemographic variables as ethnicity, gender identity and migration status should be included in further studies since these are relevant to the development of identity. Second, the assessment of identity in longitudinal studies is necessary to explore the stability of the scores in the long term, as well as how identity can change throughout time. In our study, we performed a 2-week retest within the school population but did not include participants from the clinical sample. Third, our school sample did not have an assessment on personality functioning to check their health status any more than the internalizing/externalizing symptoms on the Strength and Difficulties Questionnaire. Moreover, the assessment of our clinical sample needs to be studied in more detail, involving more participants and different diagnoses. The clinical sample was assessed solely based on the Structured Clinical Interview for Axis II Disorders in an outpatient service. Further studies in Panamanian population should involve the assessment of internalizing/externalizing dimensions and other personality inventories to confirm the diagnosis in clinical sample. A larger and more diverse sample with different diagnoses will enable to perform ROC analysis and set up a cut off score for clinical use. At last, further studies with robust methods are necessary to confirm the unidimensionality of the AIDA inventory adapted for Panamanian population and the replicability of these results in other adapted versions of the inventory, since Bifactor analyses can ignore cross loadings and inflate variance to general factor, in favor of these results.

## Conclusion

Method of culture-adapted translation and step-by-step test construction was successful. It was possible to build a version AIDA Panama with 58 items with excellent psychometric properties, equivalent to the original version of AIDA and other translated versions, Moreover, an inventory using a dimensional model as AIDA is relevant to study identity diffusion as a component of personality functioning across culture (89, 90). In this study we found that diffusion scale accounts for the majority of variance, indicating an unidimensional measure. The inventory shows convergent validity with relevant constructs as primitive defenses and psychiatric symptoms. The AIDA is a valid inventory to assess Identity functioning in Panamanian adolescent population in clinical and mental health research.

## Data Availability Statement

The raw data supporting the conclusions of this article will be made available by the authors, without undue reservation.

## Ethics Statement

The studies involving human participants were reviewed and approved by Comité de Bioética—Hospital Santo Tomás. Written informed consent to participate in this study was provided by the participants' legal guardian/next of kin. Written informed consent was obtained from the individual(s) and minor(s)' legal guardian/next of kin, for the publication of any potentially identifiable images or data included in this article.

## Author Contributions

SG designed the study, collected the data, and wrote the first draft. KG contributed to the study design, inputs and revisions on the draft, and performed statistical analyses. RD-H collaborated with statistical analyses and supplied on the draft. All authors contributed to and have approved the final manuscript.

## Funding

Universidad Católica Santa María la Antigua (SRUI-CPEI-ID-2015-2016-013) supported this research.

## Conflict of Interest

The authors declare that the research was conducted in the absence of any commercial or financial relationships that could be construed as a potential conflict of interest.

## Publisher's Note

All claims expressed in this article are solely those of the authors and do not necessarily represent those of their affiliated organizations, or those of the publisher, the editors and the reviewers. Any product that may be evaluated in this article, or claim that may be made by its manufacturer, is not guaranteed or endorsed by the publisher.
