# Supplementary material for: Psychometric Properties of a Cultural Adapted Version of the Assessment of Identity Development in Adolescence in Panama
Source: Front Psychiatry. 2022 Mar 31;13:806033. doi: 10.3389/fpsyt.2022.806033 (PMC9009042; doi:10.3389/fpsyt.2022.806033)
Supplement: Supplementary file 1 [file Table_1.docx]

Supplementary Material

**Table 1. Factor Loadings from Bifactorial Confirmatory Analysis**

|  | Unstandardized Loadings | | | | Standardized Loadings | |
| --- | --- | --- | --- | --- | --- | --- |
| LATENT VARIABLES | Estimate | Standard Error | z-value | p(>\|z\|) | Estimate | Standard Error |
| Perspectives |  |  |  |  |  |  |
| q1 | 0.508 | 0.076 | 6.649 | 0.000 | 0.508 | 0.076 |
| q5 | 0.402 | 0.069 | 5.863 | 0.000 | 0.402 | 0.069 |
| q26 | 0.633 | 0.095 | 6.678 | 0.000 | 0.633 | 0.095 |
| q58 | 0.624 | 0.074 | 8.393 | 0.000 | 0.624 | 0.074 |
| q8 | -0.189 | 0.093 | -2.034 | 0.042 | -0.189 | 0.093 |
| q33 | 0.762 | 0.082 | 9.287 | 0.000 | 0.762 | 0.082 |
| q17 | 0.429 | 0.077 | 5.590 | 0.000 | 0.429 | 0.077 |
| q27 | -0.092 | 0.071 | -1.299 | 0.194 | -0.092 | 0.071 |
| q41 | 0.420 | 0.090 | 4.648 | 0.000 | 0.420 | 0.090 |
| Relationships |  |  |  |  |  |  |
| q9 | 0.372 | 0.108 | 3.438 | 0.001 | 0.372 | 0.108 |
| q40 | 0.613 | 0.112 | 5.462 | 0.000 | 0.613 | 0.112 |
| q54 | 0.648 | 0.095 | 6.802 | 0.000 | 0.648 | 0.095 |
| q2 | 0.132 | 0.130 | 1.010 | 0.313 | 0.132 | 0.130 |
| q18 | 0.188 | 0.122 | 1.540 | 0.124 | 0.188 | 0.122 |
| q23 | 0.029 | 0.170 | 0.173 | 0.863 | 0.029 | 0.170 |
| q28 | -0.014 | 0.136 | -0.105 | 0.916 | -0.014 | 0.136 |
| q55 | 0.274 | 0.109 | 2.519 | 0.012 | 0.274 | 0.109 |
| q10 | 0.142 | 0.104 | 1.366 | 0.172 | 0.142 | 0.104 |
| q39 | 0.337 | 0.166 | 2.032 | 0.042 | 0.337 | 0.166 |
| q43 | -0.211 | 0.134 | -1.581 | 0.114 | -0.211 | 0.134 |
| Emotional Self Experience | | | | | | |
| q3 | -0.138 | 0.093 | -1.478 | 0.139 | -0.138 | 0.093 |
| q24 | -0.111 | 0.103 | -1.077 | 0.281 | -0.111 | 0.103 |
| q29 | -0.059 | 0.097 | -0.609 | 0.543 | -0.059 | 0.097 |
| q11 | 0.339 | 0.110 | 3.078 | 0.002 | 0.339 | 0.110 |
| q19 | 0.768 | 0.154 | 4.975 | 0.000 | 0.768 | 0.154 |
| q30 | 0.138 | 0.099 | 1.394 | 0.163 | 0.138 | 0.099 |
| q44 | 0.864 | 0.183 | 4.724 | 0.000 | 0.864 | 0.183 |
| Consistency |  |  |  |  |  |  |
| q12 | 0.222 | 0.140 | 1.588 | 0.112 | 0.222 | 0.140 |
| q25 | 0.010 | 0.181 | 0.058 | 0.954 | 0.010 | 0.181 |
| q31 | 0.303 | 0.158 | 1.919 | 0.055 | 0.303 | 0.158 |
| q57 | 0.199 | 0.126 | 1.577 | 0.115 | 0.199 | 0.126 |
| q4 | 0.233 | 0.112 | 2.079 | 0.038 | 0.233 | 0.112 |
| q15 | 0.166 | 0.141 | 1.176 | 0.240 | 0.166 | 0.141 |
| q45 | 0.528 | 0.152 | 3.468 | 0.001 | 0.528 | 0.152 |
| q47 | 0.371 | 0.179 | 2.076 | 0.038 | 0.371 | 0.179 |
| q13 | -0.115 | 0.129 | -0.895 | 0.371 | -0.115 | 0.129 |
| q32 | -0.373 | 0.115 | -3.242 | 0.001 | -0.373 | 0.115 |
| q56 | -0.245 | 0.147 | -1.666 | 0.096 | -0.245 | 0.147 |
| Autonomy |  |  |  |  |  |  |
| q14 | 0.154 | 0.088 | 1.761 | 0.078 | 0.154 | 0.088 |
| q21 | 0.318 | 0.096 | 3.327 | 0.001 | 0.318 | 0.096 |
| q22 | 0.690 | 0.073 | 9.451 | 0.000 | 0.690 | 0.073 |
| q34 | 0.069 | 0.098 | 0.703 | 0.482 | 0.069 | 0.098 |
| q42 | 0.404 | 0.117 | 3.466 | 0.001 | 0.404 | 0.117 |
| q46 | 0.442 | 0.134 | 3.294 | 0.001 | 0.442 | 0.134 |
| q20 | 0.627 | 0.089 | 7.031 | 0.000 | 0.627 | 0.089 |
| q38 | 0.618 | 0.090 | 6.876 | 0.000 | 0.618 | 0.090 |
| q48 | 0.239 | 0.105 | 2.280 | 0.023 | 0.239 | 0.105 |
| q53 | 0.460 | 0.100 | 4.576 | 0.000 | 0.460 | 0.100 |
| q36 | 0.120 | 0.103 | 1.158 | 0.247 | 0.120 | 0.103 |
| q50 | 0.059 | 0.106 | 0.554 | 0.579 | 0.059 | 0.106 |
| Cognitive Self Experience | | | | | | |
| q6 | 0.323 | 0.332 | 0.974 | 0.330 | 0.323 | 0.332 |
| q37 | 0.317 | 0.252 | 1.257 | 0.209 | 0.317 | 0.252 |
| q51 | 0.446 | 0.230 | 1.942 | 0.052 | 0.446 | 0.230 |
| q7 | -0.173 | 0.251 | -0.690 | 0.490 | -0.173 | 0.251 |
| q16 | 0.104 | 0.120 | 0.863 | 0.388 | 0.104 | 0.120 |
| q35 | -0.100 | 0.154 | -0.646 | 0.518 | -0.100 | 0.154 |
| q49 | 0.654 | 0.288 | 2.270 | 0.023 | 0.654 | 0.288 |
| q52 | 0.591 | 0.099 | 5.993 | 0.000 | 0.591 | 0.099 |
| General Factor Identity Diffusion | | | | | | |
| q1 | 0.385 | 0.084 | 4.589 | 0.000 | 0.385 | 0.084 |
| q5 | 0.212 | 0.075 | 2.823 | 0.005 | 0.212 | 0.075 |
| q26 | 0.258 | 0.097 | 2.670 | 0.008 | 0.258 | 0.097 |
| q58 | 0.153 | 0.094 | 1.634 | 0.102 | 0.153 | 0.094 |
| q8 | 0.734 | 0.082 | 8.978 | 0.000 | 0.734 | 0.082 |
| q33 | 0.067 | 0.093 | 0.719 | 0.472 | 0.067 | 0.093 |
| q17 | 0.458 | 0.081 | 5.677 | 0.000 | 0.458 | 0.081 |
| q27 | 0.727 | 0.074 | 9.762 | 0.000 | 0.727 | 0.074 |
| q41 | 0.258 | 0.089 | 2.903 | 0.004 | 0.258 | 0.089 |
| q9 | 0.497 | 0.083 | 6.019 | 0.000 | 0.497 | 0.083 |
| q40 | 0.532 | 0.083 | 6.440 | 0.000 | 0.532 | 0.083 |
| q54 | 0.523 | 0.075 | 7.004 | 0.000 | 0.523 | 0.075 |
| q2 | 0.388 | 0.078 | 4.964 | 0.000 | 0.388 | 0.078 |
| q18 | 0.967 | 0.080 | 12.152 | 0.000 | 0.967 | 0.080 |
| q23 | 0.604 | 0.087 | 6.974 | 0.000 | 0.604 | 0.087 |
| q28 | 0.633 | 0.085 | 7.489 | 0.000 | 0.633 | 0.085 |
| q55 | 0.482 | 0.084 | 5.739 | 0.000 | 0.482 | 0.084 |
| q10 | 0.593 | 0.085 | 6.981 | 0.000 | 0.593 | 0.085 |
| q39 | 0.532 | 0.103 | 5.148 | 0.000 | 0.532 | 0.103 |
| q43 | -0.514 | 0.083 | -6.164 | 0.000 | -0.514 | 0.083 |
| q3 | 1.000 |  |  |  | 1.000 | 1.000 |
| q24 | 0.508 | 0.097 | 5.258 | 0.000 | 0.508 | 0.097 |
| q29 | 0.832 | 0.084 | 9.873 | 0.000 | 0.832 | 0.084 |
| q11 | 0.963 | 0.079 | 12.238 | 0.000 | 0.963 | 0.079 |
| q19 | 0.770 | 0.088 | 8.732 | 0.000 | 0.770 | 0.088 |
| q30 | 0.822 | 0.075 | 10.890 | 0.000 | 0.822 | 0.075 |
| q44 | 0.789 | 0.097 | 8.149 | 0.000 | 0.789 | 0.097 |
| q12 | 0.419 | 0.085 | 4.898 | 0.000 | 0.419 | 0.085 |
| q25 | 0.754 | 0.090 | 8.423 | 0.000 | 0.754 | 0.090 |
| q31 | 0.984 | 0.079 | 12.450 | 0.000 | 0.984 | 0.079 |
| q57 | 0.501 | 0.086 | 5.797 | 0.000 | 0.501 | 0.086 |
| q4 | 0.845 | 0.069 | 12.202 | 0.000 | 0.845 | 0.069 |
| q15 | 0.842 | 0.081 | 10.404 | 0.000 | 0.842 | 0.081 |
| q45 | 0.905 | 0.086 | 10.521 | 0.000 | 0.905 | 0.086 |
| q47 | 0.706 | 0.092 | 7.654 | 0.000 | 0.706 | 0.092 |
| q13 | 1.110 | 0.070 | 15.823 | 0.000 | 1.110 | 0.070 |
| q32 | 1.183 | 0.073 | 16.105 | 0.000 | 1.183 | 0.073 |
| q56 | 0.422 | 0.097 | 4.341 | 0.000 | 0.422 | 0.097 |
| q14 | 0.925 | 0.079 | 11.757 | 0.000 | 0.925 | 0.079 |
| q21 | 0.682 | 0.080 | 8.553 | 0.000 | 0.682 | 0.080 |
| q22 | 0.644 | 0.077 | 8.415 | 0.000 | 0.644 | 0.077 |
| q34 | 0.649 | 0.079 | 8.252 | 0.000 | 0.649 | 0.079 |
| q42 | 0.497 | 0.093 | 5.322 | 0.000 | 0.497 | 0.093 |
| q46 | 0.525 | 0.101 | 5.168 | 0.000 | 0.525 | 0.101 |
| q20 | 0.768 | 0.086 | 8.975 | 0.000 | 0.768 | 0.086 |
| q38 | 0.743 | 0.089 | 8.323 | 0.000 | 0.743 | 0.089 |
| q48 | 0.142 | 0.094 | 1.499 | 0.134 | 0.142 | 0.094 |
| q53 | 0.723 | 0.087 | 8.314 | 0.000 | 0.723 | 0.087 |
| q36 | 0.343 | 0.092 | 3.722 | 0.000 | 0.343 | 0.092 |
| q50 | 0.752 | 0.086 | 8.692 | 0.000 | 0.752 | 0.086 |
| q6 | 0.541 | 0.098 | 5.504 | 0.000 | 0.541 | 0.098 |
| q37 | 0.923 | 0.077 | 11.991 | 0.000 | 0.923 | 0.077 |
| q51 | 0.787 | 0.091 | 8.647 | 0.000 | 0.787 | 0.091 |
| q7 | 0.829 | 0.086 | 9.617 | 0.000 | 0.829 | 0.086 |
| q16 | 0.711 | 0.087 | 8.178 | 0.000 | 0.711 | 0.087 |
| q35 | 1.062 | 0.070 | 15.082 | 0.000 | 1.062 | 0.070 |
| q49 | 0.316 | 0.082 | 3.841 | 0.000 | 0.316 | 0.082 |
| q52 | 0.742 | 0.091 | 8.114 | 0.000 | 0.742 | 0.091 |


